# An Unusual Case of Klebsiella pneumoniae Endocarditis

**DOI:** 10.7759/cureus.6999

**Published:** 2020-02-14

**Authors:** Syed Adeel Hassan, Ali Akhtar, Noor Ul Falah, Maham Khan

**Affiliations:** 1 Internal Medicine, Dow University of Health Sciences, Karachi, PAK; 2 Internal Medicine, Army Medical College, National University of Medical Sciences, Rawalpindi, PAK; 3 Internal Medicine, King Edward Medical University, Lahore, PAK; 4 Radiology, Armed Forces Institute of Radiology and Imaging, Rawalpindi, PAK

**Keywords:** klebsiella pneumoniae, endocarditis, pneumonia, colistin, aortic valve

## Abstract

Klebsiella pneumoniae notoriously causes life-threatening community-acquired or hospital-acquired pneumonia. In the United States, community-acquired pneumonia is a relatively common diagnosis. However, community-acquired pneumonia due to Klebsiella pneumoniae is fairly uncommon. Delayed antibiotic administration can result in bacteremia, septicemia and other systemic complications. Infective endocarditis arising as a complication of community-acquired Klebsiella pneumoniae infection has rarely been reported. Our patient is an 88-year-old diabetic female, who was admitted to our intensive care unit due to a high-grade fever, worsening dyspnea and hypotension. Chest x-ray and blood culture were conclusive for pneumonia due to Klebsiella pneumoniae. Importantly, the species was only susceptible to colistin. Furthermore, an echocardiogram revealed mobile vegetations of the non-coronary cusp of the aortic valve. Treatment with colistin resulted in clinical improvement and an uneventful discharge from the hospital. Follow up echocardiography was scheduled upon discharge to monitor the resolution of cardiac vegetations.

## Introduction

Klebsiella pneumoniae is a gram-negative bacteria belonging to the Enterobacteriaceae family. Carl Friedlander first isolated the bacterium from the lungs of patients who had died from pneumonia. Alcoholic and diabetic patients are at an increased risk of infection. The bacteria colonize the mucosal surfaces of the oropharynx and gastrointestinal tract. Higher rates of colonization have been reported in chronic alcoholics. Pneumonia caused by the bacteria can be broken down into hospital-acquired or community-acquired infections. Klebsiella pneumoniae accounts for 3%-5% of all community-acquired pneumonia cases. It is also known to be the cause in approximately 11.8% of cases in hospital-acquired pneumonia [1]. However, pneumonia and endocarditis due to Klebsiella pneumoniae infection are extremely rare [2]. We report a case of community-acquired Klebsiella pneumoniae infection which presented initially with pneumonia. It then clinically progressed to bacteremia resulting in sepsis. The bacteremia further involved the aortic valve resulting in endocarditis.

## Case presentation

An 88-year-old female was admitted to our medical intensive care unit with a two-day history of worsening dyspnea and hypotension. An initial assessment at the time of admission ruled out pulmonary embolism. On further inquiry, the patient had a history of consistent, unremitting, high-grade fever (100°F) for the past week. It was also noted that her dyspnea developed after the onset of high-grade fever. Additionally, she had also developed a mildly productive cough during the past week. Her past medical history was significant for long-standing uncontrolled diabetes and end-stage renal disease. Family members of the patient declined any history of bird exposure at home or as part of her occupation. Social history revealed that she was a non-smoker and non-alcoholic. Furthermore, she did not have any history of illicit drug abuse.

**Figure 1:**
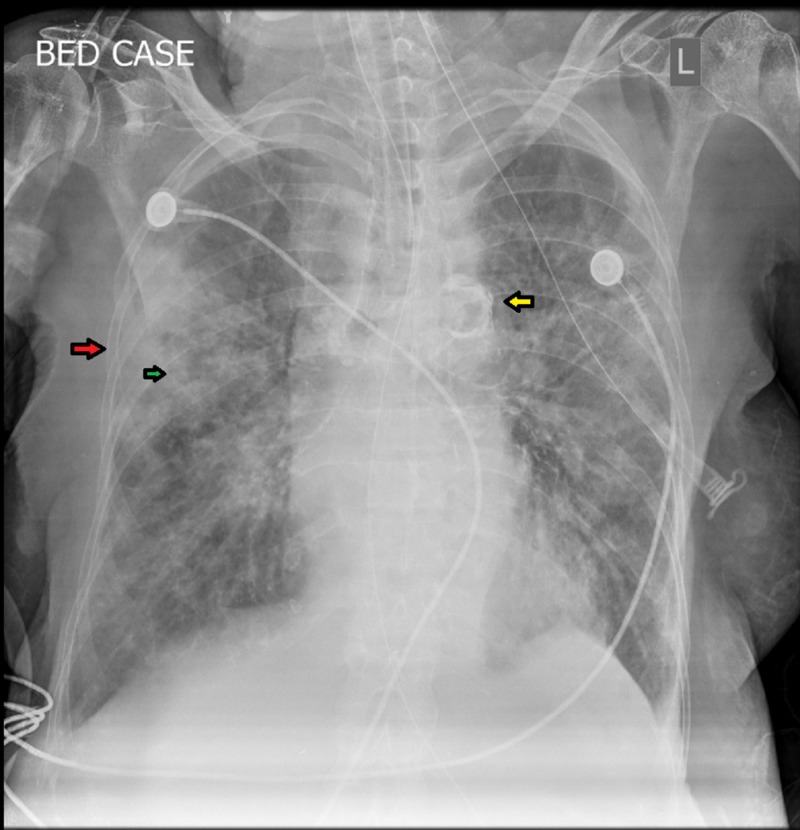
Chest X-ray depicting pathological changes of pneumonia in our patient L: Left-sided orientation of the patient Bed case: Bedside chest X-ray Widespread opacification of the respective right and left lung fields can be observed. The right upper and middle lung zone opacities have fused to impart a wedge-shaped area of opacification (red arrow). Within this wedge-shaped area, dark air-filled bronchi are being made visible by the surrounding opacification of alveoli (air bronchogram, green arrow). Physiological calcification of the aortic knuckle can also be noted (yellow arrow).

A few hours after her admission, her condition began to deteriorate. Her oxygen saturation dropped from 89% to 70%. She became severely dyspneic with a raised blood PaCO_2_ level. Based on the rapid clinical decline, the decision was taken to electively intubate her and further proceed with treatment on the grounds of sepsis. On physical examination, she was febrile (100°F), with blood pressure 80/50 mmHg and pulse 88 beats/min. Respiratory system examination was significant for bilateral basal crepitations with an expiratory wheeze. Chest x-ray revealed patchy areas of alveolar opacification in bilateral lung fields. Opacification predominantly involved the right upper and middle lung fields and left upper and lower lung fields. The diseased regions in the right lung zones collapsed to form a non-homogeneous wedge-shaped area of opacification with internal air bronchograms (Figure 1). Furthermore, physiological calcification of aortic knuckle was also evident. Based on the chest x-ray findings, a preliminary diagnosis of pneumonia was made.

**Figure 2:**
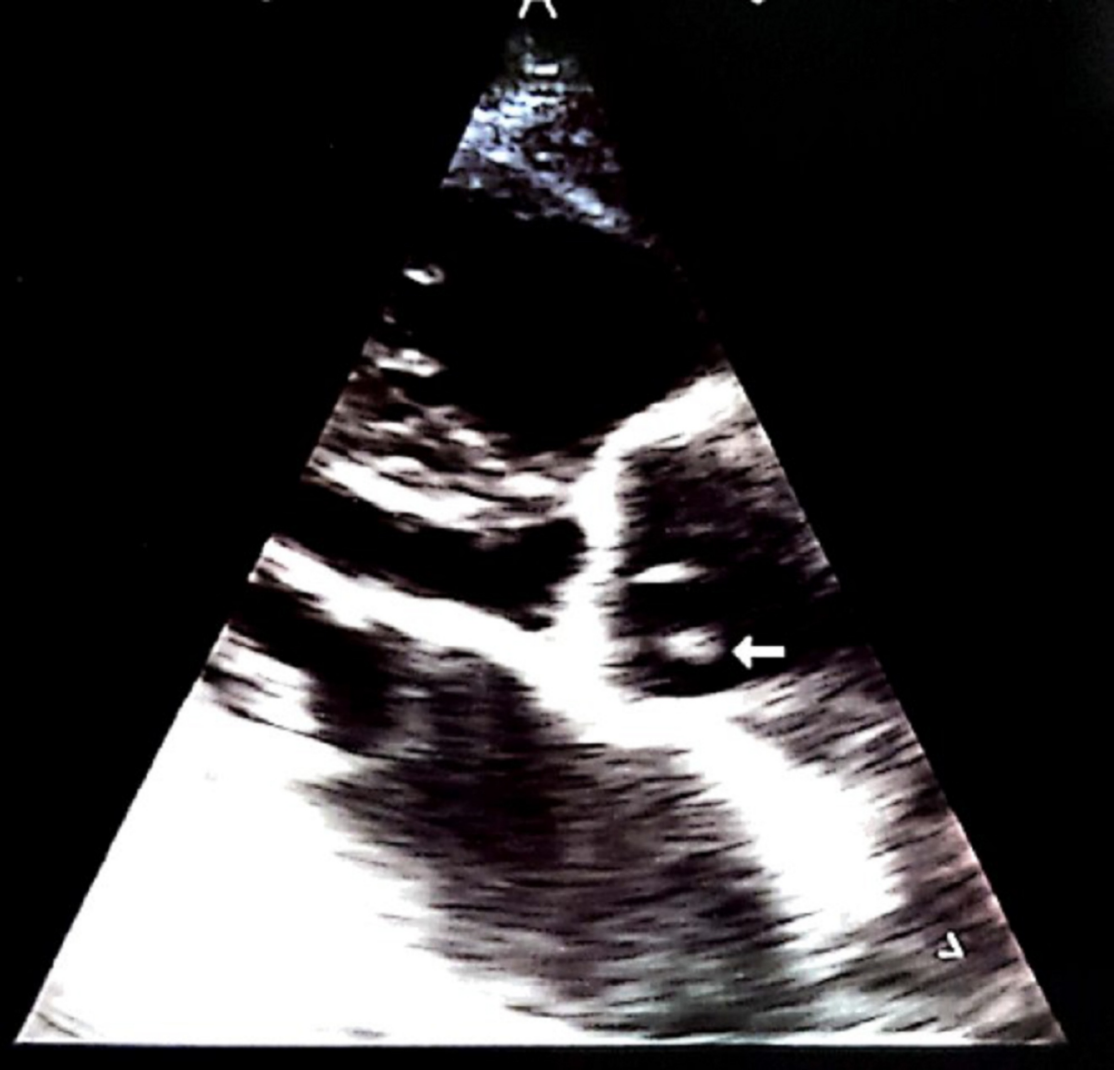
Patient's echocardiogram depicting valvular vegetations Mobile vegetations on the non-coronary cusp of the aortic valve can be seen (white arrow).

An echocardiogram revealed no obvious clots in the left ventricle. However, it was significant for mobile vegetations on the non-coronary cusp of the aortic valve (Figure 2). A grade I diastolic dysfunction with a global ejection fraction of 58% was also noted.

On the second day of admission, she suffered from a higher fever spike (102°F). This bout of fever was accompanied by leukocytosis (total leukocyte count 26.8 x 10^9^/L with a neutrophil count of 92%). The patient was started on broad-spectrum antibiotics vancomycin and cefepime. Subsequently, the result of cough sputum culture was negative. However, blood culture was positive with Klebsiella pneumoniae species which was only susceptible to colistin. The antibiotic was changed to colistin and was continued for 10 days. Thereafter, her clinical condition improved. Follow-up echocardiography was scheduled upon discharge to monitor the resolution of the vegetations.

## Discussion

Studies have identified clinical differences between hospital-acquired and community-acquired Klebsiella pneumoniae infections [3]. Community-acquired infections present initially with septic shock and respiratory failure [2]. It can also present with rapid onset fever and hemoptysis. These infections are rare in North America, Western Europe and Australia. It accounts for less than 1% of pneumonia cases requiring hospitalization [3]. The organism has the propensity to cause disease of the chest, urinary and intra-abdominal systems [4]. However, cases of bacteremia due to Klebsiella pneumoniae remain prevalent in Asia and Africa [3,5].

Klebsiella infections can metastasize to other organs and result in septic complications. Urinary tract infections and liver abscess are the most common sources of infection in Klebsiella pneumoniae bacteremia [6]. The portal of infection entry is only identified in 32% of cases [4]. Other infection entry routes such as vascular, oral or lung have rarely been reported [4]. Predisposing factors for infection include liver cirrhosis, hepatobiliary diseases, diabetes mellitus, asplenia, neoplasia, chronic alcoholism and corticosteroids [4,6]. In patients with community-acquired Klebsiella infections, diabetes mellitus is the most commonly reported predisposing factor [6]. Endocarditis due to Klebsiella pneumoniae infection accounts for 5% of all cases of endocarditis [5]. A review of 50 cases indicated that the aortic valve is most commonly involved, followed by the mitral valve [2,5]. The mortality rate for such cases is more than 50% [5]. The mitral valve tends to be involved when bacteremia due to a pyogenic liver abscess tends to predominate [2]. Significant risk factors for mortality include septic shock, respiratory failure, lung infection, leukopenia, thrombocytopenia and inappropriate antimicrobial therapy [2,6].

Consistent with community-acquired Klebsiella pneumoniae infections, our patient presented with a toxic clinical presentation comprising of respiratory difficulty and septic shock-like presentation. The initial fulminant pneumonia likely acted as a source of progression to bacteremia. This resulted in clinical deterioration due to the development of septic shock. Pneumonia was also a nidus for the development of aortic valve vegetation in our case. Also consistent was the presence of long-standing diabetes mellitus as a predisposing risk factor and aortic valve involvement. As opposed to the findings of high mortality in literature, our patient had a good clinical outcome with colistin. A delay in seeking medical care and the administration of appropriate antibiotics could be responsible for the rapid clinical progression to septic shock and the development of endocarditis. 

## Conclusions

Community-acquired Klebsiella pneumoniae infections seldom occur. However, cases of bacteremia are still common in Asia. Pneumonia can act as a source of clinical progression to bacteremia. As a consequence, complications such as septic shock and endocarditis ensue. In community-acquired cases, the aortic valve tends to be the most commonly involved heart valve. Commonly associated predisposing factors for infection include chronic alcoholism and diabetes mellitus. This case highlights the severity and clinical consequences of community-acquired Klebsiella pneumoniae infections. It also indicates the need for the early management of the patient to avoid the development of bacteremic clinical consequences.
